# *Saccharomyces boulardii* CNCM I-745 protects against small intestinal injury in a model of gliadin-peptide-induced sterile inflammatory enteropathy

**DOI:** 10.3389/fimmu.2026.1846961

**Published:** 2026-07-06

**Authors:** Aluminé Lezcano, Emanuel Miculan, Geronimo Ducca, Carolina Ruera, María Luz Iribarren, Philippe Tixador, Xavier Roux, Fernando Gabriel Chirdo

**Affiliations:** 1 Departamento de Ciencias Biológicas, Facultad de Ciencias Exactas, Instituto de Estudios Inmunológicos y Fisiopatológicos - IIFP (UNLP-CONICET), Universidad Nacional de La Plata, La Plata, Argentina; 2 Microbiology Department, Biocodex Simplified Joint-Stock Company (SAS), Compiegne, France

**Keywords:** cell death, intestinal inflammation, probiotics, Saccharomyces boulardii, small intestine, sterile inflammation

## Abstract

**Introduction:**

Inflammation of the small intestine can be caused by infections, immune-mediated disorders (such as celiac disease) and medications, leading to malabsorption, chronic diarrhea, and subclinical alterations including increased gut permeability and systemic inflammation. It has been suggested that probiotics can restore barrier integrity and reduce inflammation. The objective of the present study was to evaluate the effects of the probiotic yeast *Saccharomyces boulardii* CNCM I-745 (*Sb*) in a mouse model of sterile, inflammation-induced enteropathy based on intragastric administration of the gliadin peptide p31-43. Challenge with this peptide induces histological changes in the small intestinal mucosa, causes an elevation in inflammatory markers, and promotes cell death.

**Methods:**

The mice were challenged with a single dose of p31-43 or multiple doses over a three-week period, with or without *Sb* coadministration. Intestinal damage was assessed with regard to histologic features, mRNA gene expression (using RT-qPCR assays), caspase-1 activation (using Western blots), and cell death (using TUNEL reaction).

**Results and discussion:**

Compared with mice treated with single or repeated doses of p31-43 alone, concomitant *Sb* treatment was associated with significantly better histological parameters, with restoration of the villus-height-to-crypt-depth ratio and less intraepithelial lymphocyte infiltration. *Sb* prevented the p31-43–induced upregulation of *Cxcl10* expression. A Western blot analysis demonstrated that *Sb* attenuated caspase-1 activation, which indicated the suppression of p31-43–induced inflammasome activation. Furthermore, *Sb* prevented the increase in cell death observed following p31-43 treatment. No differences in goblet cell numbers were observed. However, *Muc2* mRNA expression was upregulated following p31-43 treatment alone or in combination with *Sb*. Similarly, the analysis of the expression of markers associated with epithelial cell proliferation and differentiation (*Atoh1* and *Hes1*) suggested a potential shift toward enhanced differentiation of secretory epithelial cells. In conclusion, *Saccharomyces boulardii CNCM I-745* significantly attenuated mucosal damage, inflammation, and cell death in a murine model of small intestinal inflammation. These results suggest that *Sb* has the potential to prevent or alleviate inflammation-induced damage to the small intestine and underscore the importance of probiotic yeasts and the mycobiome in modulating host responses in the gut. Clinical evaluation in celiac disease and related disorders is now warranted.

## Introduction

Probiotics have emerged as promising agents for the treatment of a variety of inflammatory conditions. These viable microorganisms exert beneficial effects on multiple aspects of human health and disease (1). In particular, several studies have evidenced beneficial effects of probiotic administration in patients with intestinal inflammatory disorders, such as inflammatory bowel disease and irritable bowel syndrome (2, 3). The probiotics’ mechanisms of action include the production of microbial metabolites (such as short-chain fatty acids and mainly butyrate), the upregulation of defensin and mucin-2 expression, and the downregulation of pro-inflammatory cytokine expression (4).

Various probiotic species and strains have been used to treat intestinal inflammatory disorders. For example, *Escherichia coli* Nissle 1917 has been used in the management of ulcerative colitis (5), while *Bifidobacterium infantis* 35624 has shown benefits in patients with irritable bowel syndrome (6). *Saccharomyces boulardii* has also been employed in the treatment of intestinal inflammatory conditions. This probiotic yeast supports gut health through several mechanisms (7). Firstly, *S. boulardii* exerts direct anti-inflammatory effects on intestinal epithelial cells by modulating key signaling pathways, including nuclear factor-kappa B (NF-κB) and mitogen-activated protein kinases (MAPKs) (8). Secondly, the yeast has well-characterized beneficial actions in the probiotic treatment of intestinal infections, including the production of antimicrobial peptides, the ability to bind pathogenic bacteria (such as enterohemorrhagic *E. coli* and *Salmonella typhimurium*), and the ability to neutralize toxins secreted by pathogens such as *Clostridioides difficile* (9, 10). *Saccharomyces boulardii* CNCM I-745 (hereafter referred to as *Sb*) is the most widely used strain, and its efficacy and safety have been demonstrated in many randomized clinical trials (11).

The gut immune system is complex, and a number of mechanisms appear to account for the effects of probiotics in this context. However, most mechanistic studies of probiotic strains have focused on inflammatory diseases of the colon or on intestinal infections, and far less attention has been given to the small intestine and to non-infectious conditions.

Celiac disease (CeD) is a highly prevalent, multifactorial, immune-mediated enteropathy that primarily affects the proximal small intestine. This chronic inflammatory condition is driven by an exacerbated, uncontrolled immune response to dietary proteins from wheat, barley, and rye, collectively referred to as gluten in genetically predisposed individuals. This immune response can severely damage the proximal small intestine, as characterized histologically by a low villus-height-to-crypt-depth (V/C) ratio and an elevated number of intraepithelial lymphocytes (IELs) (12). At present, an animal model that fully reproduces the pathogenesis of CeD is lacking.

With a view to evaluating the inflammatory environment in the proximal small intestine, we developed a mouse model based on the intragastric administration of p31-43, a synthetic, gliadin-derived, 13mer peptide that reproduces several histological features of CeD (13). p31–43 does not bind human leukocyte antigen (HLA) class II molecules and does not induce classical T cell activation. Nevertheless, the results of *in vitro* and *in vivo* assays demonstrated that p31–43 produces toxic effects and triggers strong inflammatory responses (14). We have previously demonstrated *in vivo* that intragastric administration of p31–43 results in damage to the proximal small intestinal, as characterized by a low V/C ratio and elevated IEL infiltration (13). Furthermore, our model revealed the activation of distinctly regulated cell death pathways (15), with marked induction of the NLRP3 inflammasome and the production of inflammatory mediators (13, 15, 16).

Although various probiotics have demonstrated beneficial effects in the context of gut inflammation, published data on the corresponding mechanism(s) of action in the proximal small intestine are scarce. The objective of the present study was therefore to investigate the role of *Sb* in modulating the inflammatory response in two mouse models of sterile inflammation in the proximal small intestine.

## Materials and methods

### Reagents and the probiotic

The p31–43 gliadin-derived peptide (amino acid sequence: LGQQQPFPPQQPY) was synthesized (purity: >95%) by GeneCust (Luxembourg). A solution of p31–43 was prepared by resuspending the lyophilized peptide in sterile phosphate-buffered saline (PBS).

*Saccharomyces boulardii* CNCM I-745 (*Sb*) was provided by Biocodex (Gentilly, France). For probiotic treatment, the *Sb* suspension was freshly prepared daily at a concentration of 300 mg/mL in sterile water.

### Mice

Eight weeks old female C57BL/6 wild-type mice were purchased from the Animal Care Facility at the Faculty of Veterinary Sciences, National University of La Plata (La Plata, Argentina). All mice were housed under specific pathogen-free conditions and were allowed *ad libitum* access to autoclaved food and water. Mice were euthanized by cervical dislocation performed by trained personnel. All experimental procedures complied with local regulatory and ethical guidelines and were approved by the Institutional Animal Care and Use Committee of the Faculty of Exact Sciences, National University of La Plata (reference: 007-44-23).

### Experimental model of intestinal inflammation and treatment with *Sb*

**Figure 1:**
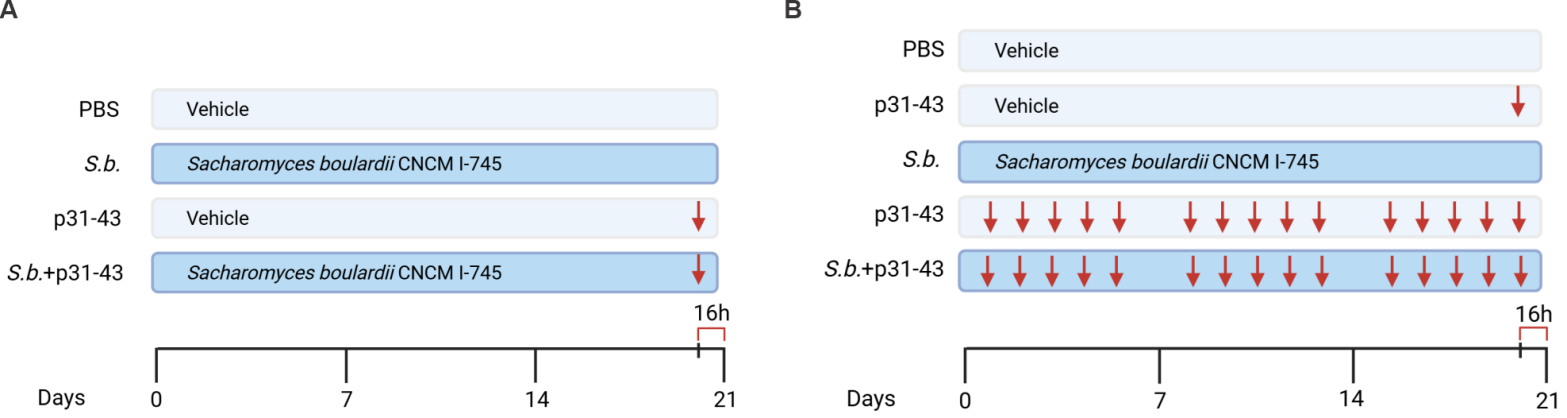
The experimental design. **(A)** The preventive (single p31–43 challenge) protocol. **(B)** The therapeutic (repeated p31–43 challenges) protocol. In both designs, the red arrows indicate intragastric administration of p31-43.

Two protocols were applied: a preventive protocol and a therapeutic protocol. Our preventive protocol consisted of treating mice daily by intragastric administration (IG) using a curved oral gavage needle (22G, 3, 8 cm) with *Sb* (3 g/kg/day) or vehicle five times a week for three weeks (**Figure 1A**). Inflammation was induced by one IG administration of gliadin peptide p31-43 (40 µg/mouse) 16 hours before euthanasia. To analyze gene expression by RT-qPCR, the same experimental protocol was followed; however, mice were euthanized 4 h after the IG administration of p31–43.

In the therapeutic protocol, mice were treated by IG with gliadin peptide p31-43 (40 μg/mouse) or vehicle (PBS) five times a week for three weeks (**Figure 1B**). As in the preventive protocol, mice were treated by IG with *Sb* (3 g/kg/day) or vehicle five times a week for three weeks. In addition, a group of mice received a single IG dose of p31–43, following the same procedure described above, and were euthanized 16 h later to allow comparison with the repeated-challenge protocol.

For both protocols, five mice were assigned randomly to each experimental group.

### Histological evaluation

Sections of proximal small intestine were fixed in 4% w/v formaldehyde for 24 h, embedded in paraffin, prepared as 5 µm sections, and stained with hematoxylin and eosin (H&E) or alcian blue for histological evaluation under a Nikon Eclipse Ti microscope. Images were analyzed using QuPath software (version 0.4.3, https://qupath.github.io/). At least 30 V/C ratios were assessed in each mouse, while IELs were counted in 10 randomly chosen villus tips and expressed as IELs/100 enterocytes. Lastly the number of goblet cells was counted on alcian-blue-stained sections and expressed as alcian-blue-positive cells/villus.

### Western blot analysis

Small intestine samples from individual mice were resuspended in 300 µl of RIPA cell lysis buffer (Cat# ab156034, Abcam) supplemented with protease inhibitor cocktail and EDTA (Cat# ab271306, Abcam). Protein extracts were separated by SDS-PAGE on 12.5% gels and transferred into 0, 45 µm nitrocellulose membranes (Cat# 10600003, Amersham Cytiva). The membranes were blocked with 5% w/v fat-free dry milk in Tris-buffered saline pH 8 (TBS) at 37°C for 1 h and incubated at 4°C overnight with the primary antibody anti-caspase-1 (1/800 Cat# Sc-56036, Santa Cruz Biotechnology), anti-caspase-3 (Cat# nb100-56113, Novus) or anti-β-actin (1/1500 Cat# ab8227, Abcam). After washing with Tris-buffered saline 0.1% v/v Tween 20, membranes were incubated with horse-radish-peroxidase-conjugated secondary goat anti-mouse antibody (1/2500 Cat# ab6789, Abcam) or horse-radish-peroxidase-conjugated secondary goat anti-rabbit antibody (1/2000 Cat# 1706515, BioRad) at 37°C for 1 h, and visualized using the enhanced chemiluminescent reagent (RPN2232, Amersham Cytiva) in a C-Digit Blot Scanner LI-COR. The relative levels of the target protein were determined using ImageStudio software (LI-COR Biosciences, Lincoln, NE, USA).

### RNA extraction and quantitative real-time PCR

Small intestine samples from mice were stored in RNA Later Solution (Cat# AM720, Invitrogen by Thermo Fisher Scientific) at -20°C for 4 h and then removed and stored at -80°C. Total RNA was extracted from the samples using TRIzol reagent (Cat# 15596026, Invitrogen by Thermo Fisher Scientific), and the concentration of RNA was determined with Varioskan LUX (Thermo Fisher Scientific). cDNA was synthesized from 2 µg RNA using M-MLV reverse transcriptase (Invitrogen) and real-time PCR was performed using SYBR green PCR Master mix (BioRad) on a CFX Opus 96 cycler (BioRad). The amplification protocol consisted of 40 cycles, using the primers listed in **Table 1**. To normalize expression levels, *Hprt* was used as an internal control. All results were expressed as the fold increase for each treatment vs the means of *Sb* treated samples at each time point, using the 2^−△△Ct^ method.

**Table 1:** Nucleotide sequences of forward and reverse primers.

Target gene	Forward (5’ to 3’) primer	Reverse (5’ to 3’) primer
*Hprt*	GTTAAGCAGTACAGCCCCAAA	AGGGCATATCCAACAACAAACTT
*Areg*	GGTGGTGACATGCAATTGTC	CATCCTCGCTGTGAGTCTTC
*Atoh1*	CAGGGTGAGCTGGTAAGGAG	GCCAAGCTCGTCCACTACA
*Cldn2*	TGAACACGGACCACTGAAAG	TTAGCAGGAAGCTGGGTCAG
*Cldn4*	CGAGCCCTTATGGTCATCAGCA	ATGCTTGCCACGATGAACACGG
*Cxcl10*	ATAGGGAAGCTTGAAATCATCC	TTCATCGTGGCAATGATCTC
*Defα5*	TCAAAAAAGCTGATATGCTATTG	AGCTGCAGCAGAATACGAAAG
*Hes1*	AGTGTCACCTTCCAGTGGCT	TGGGCTAGGGACTTTACGGG
*Ifnβ1*	GCCTTTGCCATCCAAGAGATGC	ACACTGTCTGCTGGTGGAGTTC
*Muc2*	TGCCCACCTCCTCAAAGAC	TAGTTTCCGTTGGAACAGTGAA
*Reg3γ*	CCTTCCTCTTCCTCAGGCAAT	TAATTCTCTCTCCACTTCAGAAATCCT

### Terminal deoxynucleotidyl transferase dUTP nick end labeling (TUNEL) assay

Samples of small intestine were fixed in 4% w/v formaldehyde for 24 h at room temperature, dehydrated in graded ethanol, embedded in paraffin, prepared as 5 µm sections on slides (using the flotation technique), and dried at 65 °C for 45–60 min. After deparaffinization and rehydration, the sections were treated with the DeadEnd™ Fluorometric TUNEL System (Cat# G3250, Promega) according to the manufacturer’s instructions. Nuclear staining was performed with propidium iodide (1 μg/mL, Cat# P4170-10MG, Sigma). After mounting (Cat# S3023, Dako Cytomation), sections were examined using an Eclipse Ti fluorescence microscope (X-Cite Series 120Q light source), and images were acquired with a Digital Sight DS-Ri1 camera and NIS-Elements software (all from Nikon). TUNEL-positive cells were counted using ImageJ software, after calibration of the image to measure cell density in the *lamina propria*. The results were expressed as TUNEL-positive cells/μm².

### Statistical analysis

Statistical analysis was performed with GraphPad Prism software (version 9.0, GraphPad Software LLC, Boston, MA, USA). In pairwise comparisons, an unpaired Student’s t-test was used for normally distributed variables, whereas the Mann– Whitney test was used for variables with a non-normal distribution. The threshold for statistical significance was set to p < 0.05. Data are reported as the mean ± standard error of the mean (SEM).

## Results

### A preventive protocol of *Saccharomyces boulardii* CNCM I-745 administration mitigates the histological damage induced by a single dose of p31-43

**Figure 2:**
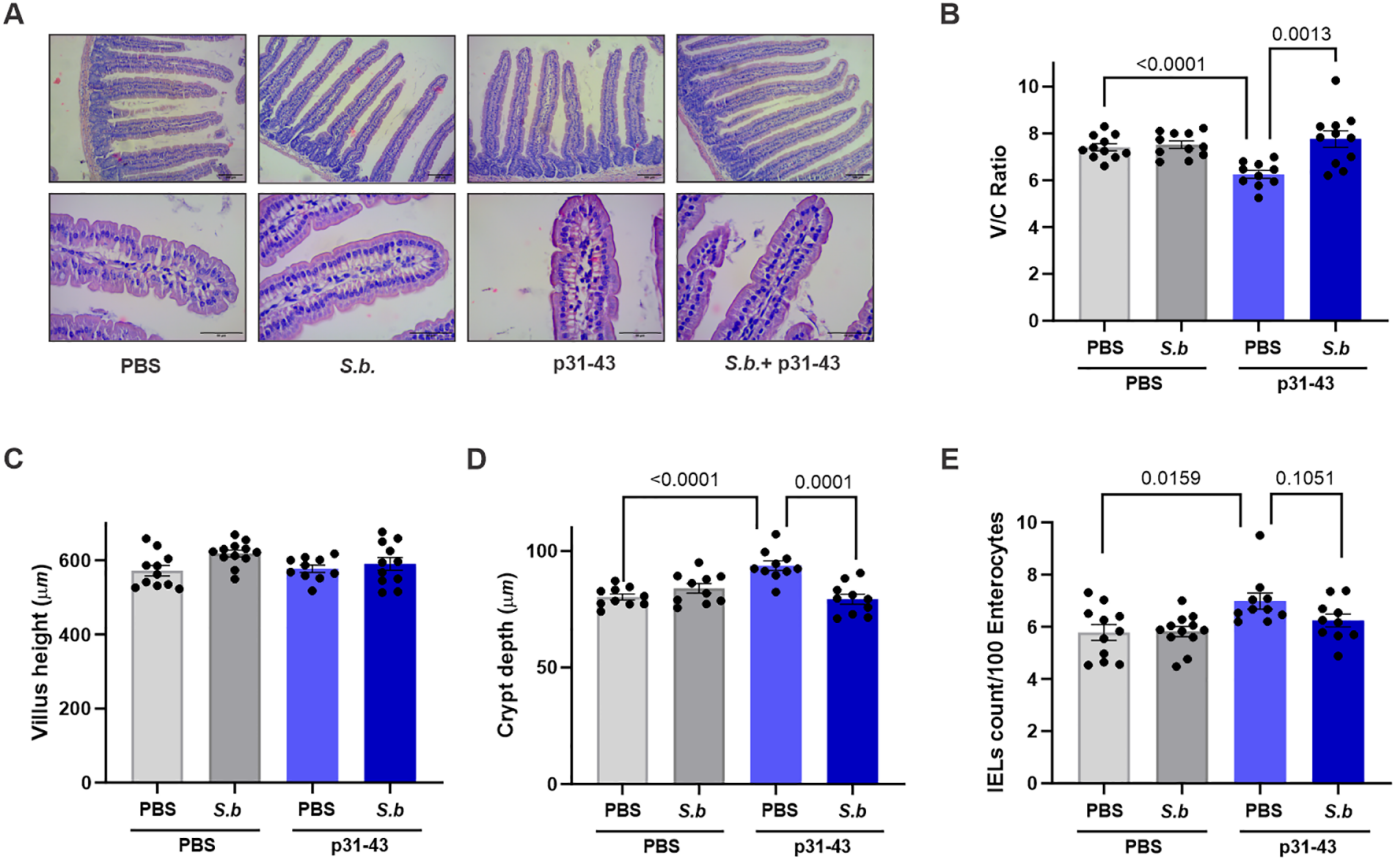
Histological analysis of the proximal small intestine in the single-challenge p31–43 model. **(A)** Representative H&E-stained sections of proximal small intestine after administration of each studied group. Upper panel: section imaged at x10 magnification, for assessment of the villus-to-crypt (V/C) ratio. Lower panel: High-magnification image (x40) for counting intraepithelial lymphocytes (IELs). **(B)** Ratio between villus height and crypt depth (V/C), **(C)** Villus height, **(D)** Crypt depth, **(E)** The mean ± SEM number of intraepithelial lymphocytes (IELs) per 100 enterocytes 16 h after p31–43 challenge. Data are from two independent experiments. Each dot represents an individual mouse (N = 11 mice). Results displayed in B, C and D were analyzed by Student´s unpaired t-test. Results displayed in E were analyzed by Mann– Whitney test. The threshold for statistical significance was set to p < 0.05.

To evaluate whether a three-week preventive protocol of *Sb* administration can mitigate the damage induced by a single dose of p31-43, histological analysis (the V/C ratio and the number of IELs) of proximal small intestine segments was performed 16 hours after p31–43 administration (**Figure 2A**). As expected, mice treated with p31–43 alone showed a lower V/C ratio (primarily due to significantly greater in crypt depth), indicating an epithelial repair response. Remarkably, preventive treatment with *Sb* was significantly associated with a healthy V/C ratio (p = 0.0013) and healthy crypt depths (p = 0.0001) (**Figures 2B D**, respectively).

IEL infiltration – a key feature of the inflammatory response - was accentuated after the administration of p31-43. The preventive *Sb* protocol tended to mitigate this effect, since the IEL count was similar to those in control (PBS-treated) groups not challenged with p31-43 (**Figure 2E**). The group treated with *Sb* alone did not differ significantly from the PBS-treated group.

Hence, administration of *Sb* either prevented the mucosal damage induced by p31–43 or was associated with a recovery of a normal histologic profile following p31–43 challenge.

### *Saccharomyces boulardii* CNCM I-745 modulates gene expression of proinflammatory and tissue repair markers in the single-challenge p31–43 model

**Figure 3:**
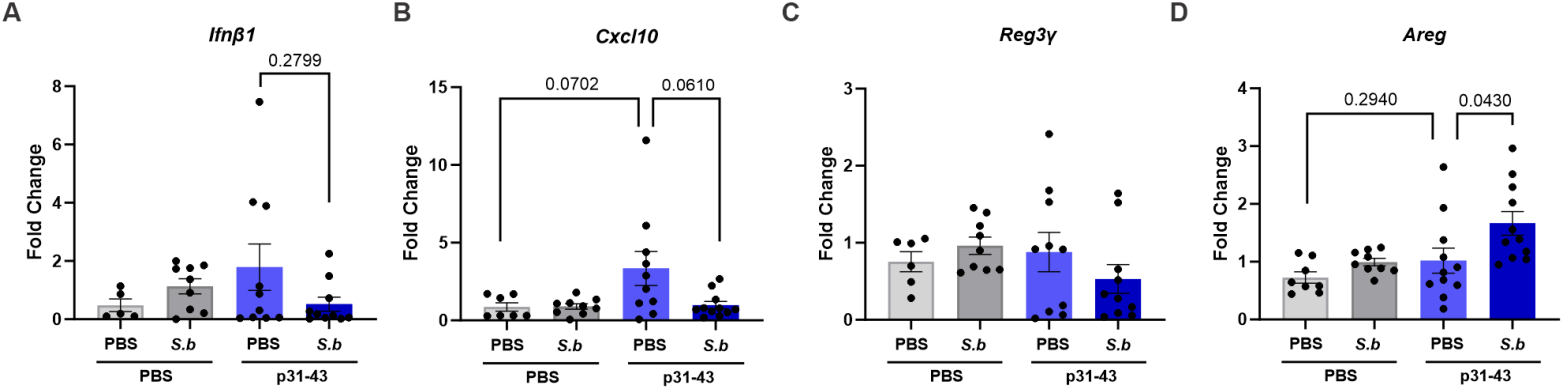
mRNA expression of genes associated with inflammatory and tissue-repair responses in the single-challenge p31–43 model. Real-time qPCR analysis of *Ifnβ1* **(A)**, *Cxcl10* **(B)**, *Reg3γ* **(C)** and *Areg* **(D)** mRNA expression 4 h after p31–43 challenge. All values were normalized against those of the housekeeping gene *Hprt*, and all results were expressed as a normalized fold increase relative to the *Sb*-treated group, using the 2^-△△Ct^ method. Each dot represents an individual mouse. Data are from two independent experiments; N = 5–11 mice per group. The results are expressed as the mean ± SEM. Results displayed in A and B were analyzed by Mann– Whitney test. Results displayed in C and D were analyzed by Student´s unpaired t-test. The threshold for statistical significance was set to p < 0.05 in Student’s unpaired t-test.

To determine whether the histological changes correlate with the expression of inflammatory genes, RT-qPCR was performed on total RNA extracted from small intestinal samples collected 4 hours after p31–43 administration. Since most pro-inflammatory markers exhibit transient upregulation, this study was performed at an earlier time point (4 h) than the other analyses (16 h). The expression levels of the inflammation markers *Ifnβ1* and *Cxcl10* were analyzed. In mice having received a preventive *Sb* protocol before challenge with p31-43, the level of *Ifnβ1* expression was lower (albeit not significantly) than in non-treated mice (**Figure 3A**). However, expression of *Cxcl10* was significantly lower (**Figure 3B**, p = 0.061) in mice receiving the preventive *Sb* protocol.

The mRNA expression level of the antimicrobial peptide *Reg3γ* did not differ significantly from one experimental group to another (**Figure 3C**). Interestingly, the gene coding for amphiregulin (*Areg*) was upregulated only in mice that received *Sb* pretreatment and were subsequently challenged with p31-43. *Areg* is known to have a key role in tissue repair mechanisms (particularly in the gut and lungs) through the promotion of epithelial cell proliferation and thus the integrity of the epithelial barrier (**Figure 3D**).

In summary, gene expression profiling 4 h after the p31–43 challenge revealed an activated inflammatory response, coupled with a tissue-repair program induced by *Sb*. Notably, administration of probiotic *Sb* normalized the expression level of the pro-inflammatory gene *Cxcl10*.

### *Saccharomyces boulardii* CNCM I-745 blocked the cell death induction promoted by a single administration of p31-43

**Figure 4:**
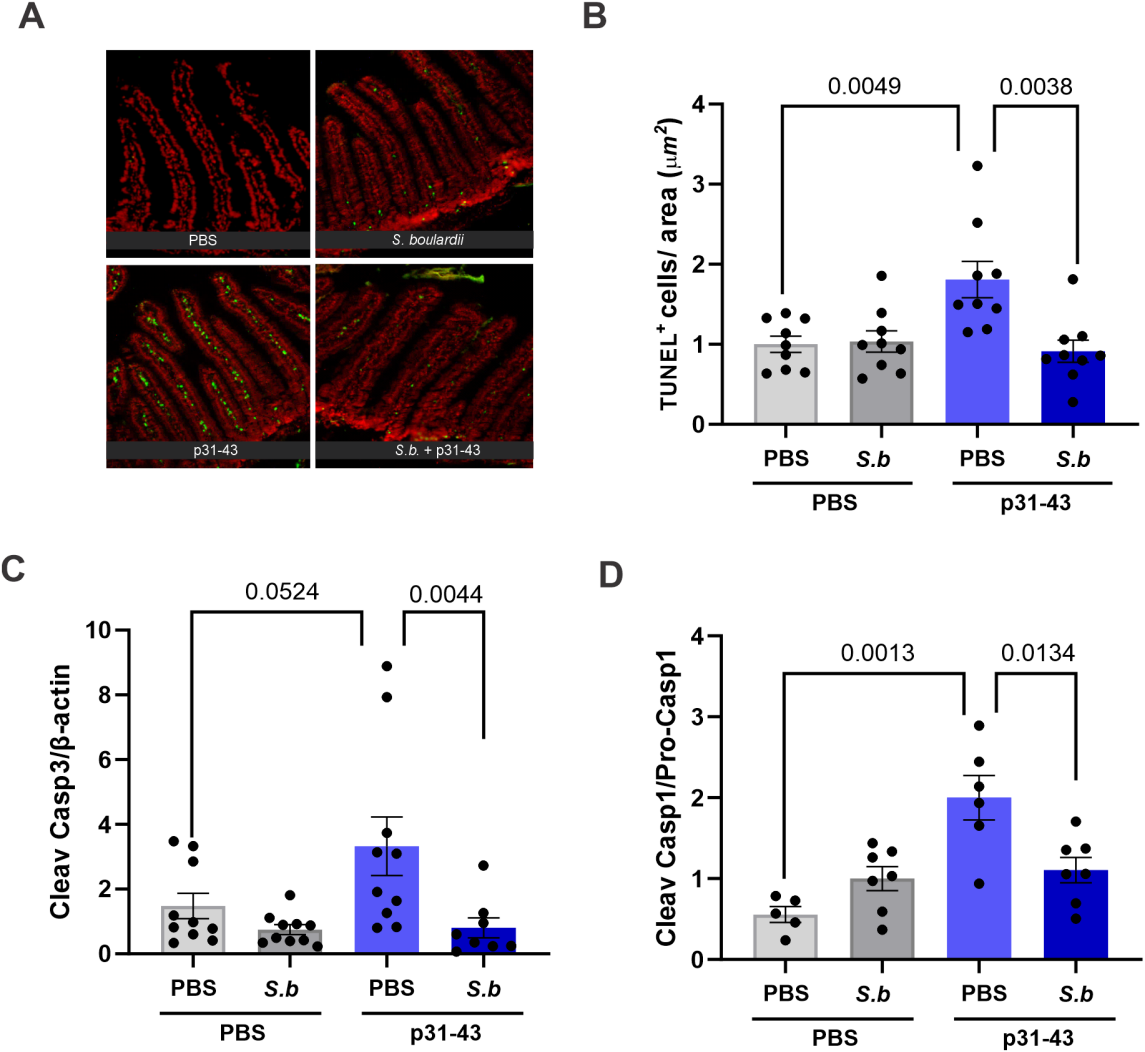
Cell death in the proximal small intestine in the single-challenge p31–43 model. **(A)** Representative images of TUNEL-stained sections of proximal small intestine. Propidium iodide staining for nuclei (red) in TUNEL-positive cells (green) **(B)** The mean ± SEM TUNEL-positive cell counts per µm^2^ of *lamina propria* (normalized). Data are from two independent experiments N = 9 mice. **(C)** Western blot analysis of cleaved caspase-3 quantification for protein extract of small intestinal fragments of individual mice. Results are expressed as the mean ± SEM ratio between band intensities for cleaved caspase-3 and β-actin N = 8–10 mice **(D)** Western blot analysis of cleaved caspase-1 and pro-caspase-1 quantification for protein extract of small intestinal fragments of individual mice. Results are expressed as the mean ± SEM ratio between band intensities for cleaved caspase-1 and pro-caspase-1. N = 5–8 mice. Results displayed in B and C were analyzed by Student´s unpaired t-test. Results displayed in D were analyzed by Mann– Whitney test. The threshold for statistical significance was set to p < 0.05 in Student’s unpaired t-test.

We next sought to determine whether the mucosal damage produced by p31–43 challenge was accompanied by a greater level of cell death (as assessed in the TUNEL assay). As expected, administration of p31–43 resulted in a significantly greater TUNEL-positive cell count in small intestine samples. Notably, pretreatment with *Sb* effectively prevented p31-43-induced cell death (p = 0.0038) (**Figure 4B**). The group treated with *Sb* alone in the absence of p31–43 challenge showed no significant differences versus the control (PBS) group. We also used Western blots to evaluate caspase-3 activation as a marker of apoptosis in protein extracts from the small intestine. Although a greater level of cleaved caspase-3 was detected in the group treated with p31-43 (**Figure 4C**), a preventive *Sb* protocol was associated with a significantly lower level (p = 0.004). This suggests that *Sb* pretreatment was effective in controlling the induction of apoptosis (**Figure 4C**). To assess activation of the inflammasome (a key element in the pyroptosis pathway), caspase-1 was evaluated on Western blots of small intestine extracts. The cleaved caspase-1 to procaspase-1 ratio (indicative of caspase-1 activation) was significantly greater in samples from the p31-43-treated mice than in samples from the PBS control group (**Figure 4D**). Remarkably, mice pretreated for three weeks with *Sb* showed a significantly lower level of inflammasome activation (p = 0.0134). The group treated with *Sb* alone did not differ significantly from the PBS control group.

Taken as a whole, these findings suggest that a preventive *Sb* administration protocol helps to mitigate the induction of both apoptosis and pyroptosis triggered by p31–43 challenge.

#### Treatment with *Saccharomyces boulardii* CNCM I-745 prevents the histological damage induced by multiple p31–43 challenges

**Figure 5:**
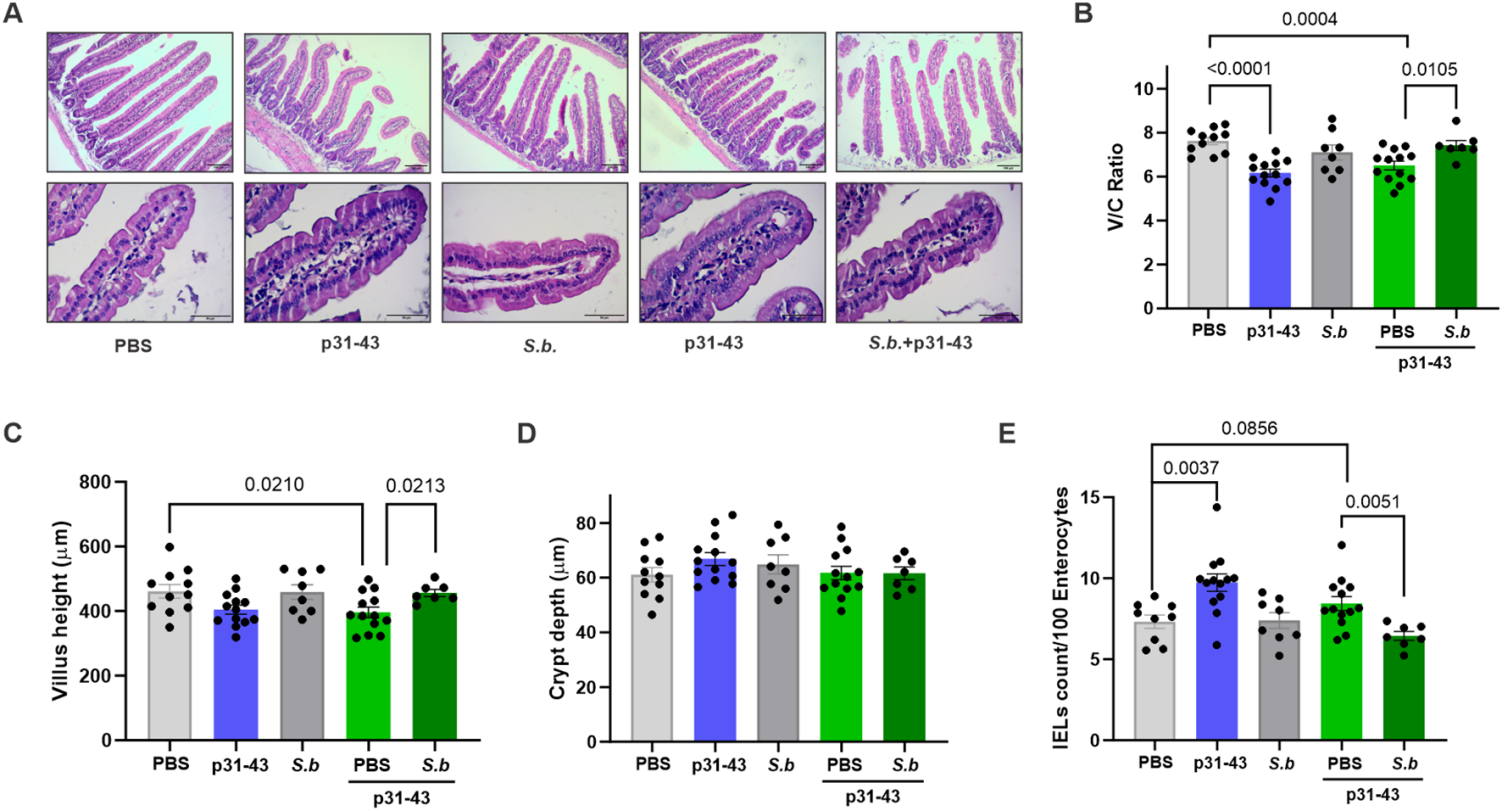
Histological analysis of the proximal small intestine in the multiple-challenge p31–43 model. **(A)** Representative H&E-stained sections of proximal small intestine after administration of each studied group. Upper panel: section imaged at x10 magnification used to assess the villus-to-crypt (V/C) ratio. Lower panel: High-magnification image (x40) used to quantify intraepithelial lymphocytes (IELs). **(B)** Ratio between villus height and crypt depth (V/C), **(C)** Villus height, **(D)** Crypt depth, **(E)** The mean ± SEM number of intraepithelial lymphocytes (IELs) per 100 enterocytes 16 h after p31–43 challenge. Data are from three independent experiments, N = 7–13 mice. The threshold for statistical significance was set to p < 0.05 in Student’s unpaired t-test.

To model a chronic inflammatory condition, mice were treated with five doses of p31–43 per week for three weeks (**Figure 1B**). The V/C ratio was significantly lower after repeated administrations of p31–43 than in the control group and was comparable to that observed following a single p31–43 challenge (**Figure 5B**). The lower V/C ratio was primarily due to villus shortening, with no significant difference in crypt depth. Remarkably, in the group treated simultaneously with *Sb* and repeated p31–43 challenges, significant normalization of the V/C ratio was observed (p = 0.0105); this was mainly due to restoration of the villus height (p = 0.0213), (**Figures 5B, C**, respectively). The group treated with *Sb* alone did not differ significantly from the PBS control group. Administration of *Sb* resulted in the significant normalization of the IEL count (p = 0.0051) (**Figure 5E**).

These findings indicate that treatment with *Sb* effectively attenuated the histopathological alterations in a model of chronic inflammation induced by repeated administration of p31-43.

#### *Saccharomyces boulardii* CNCM I-745 treatment modulated the impairments in mucus production and goblet cell differentiation induced by repeated p31–43 administration

**Figure 6:**
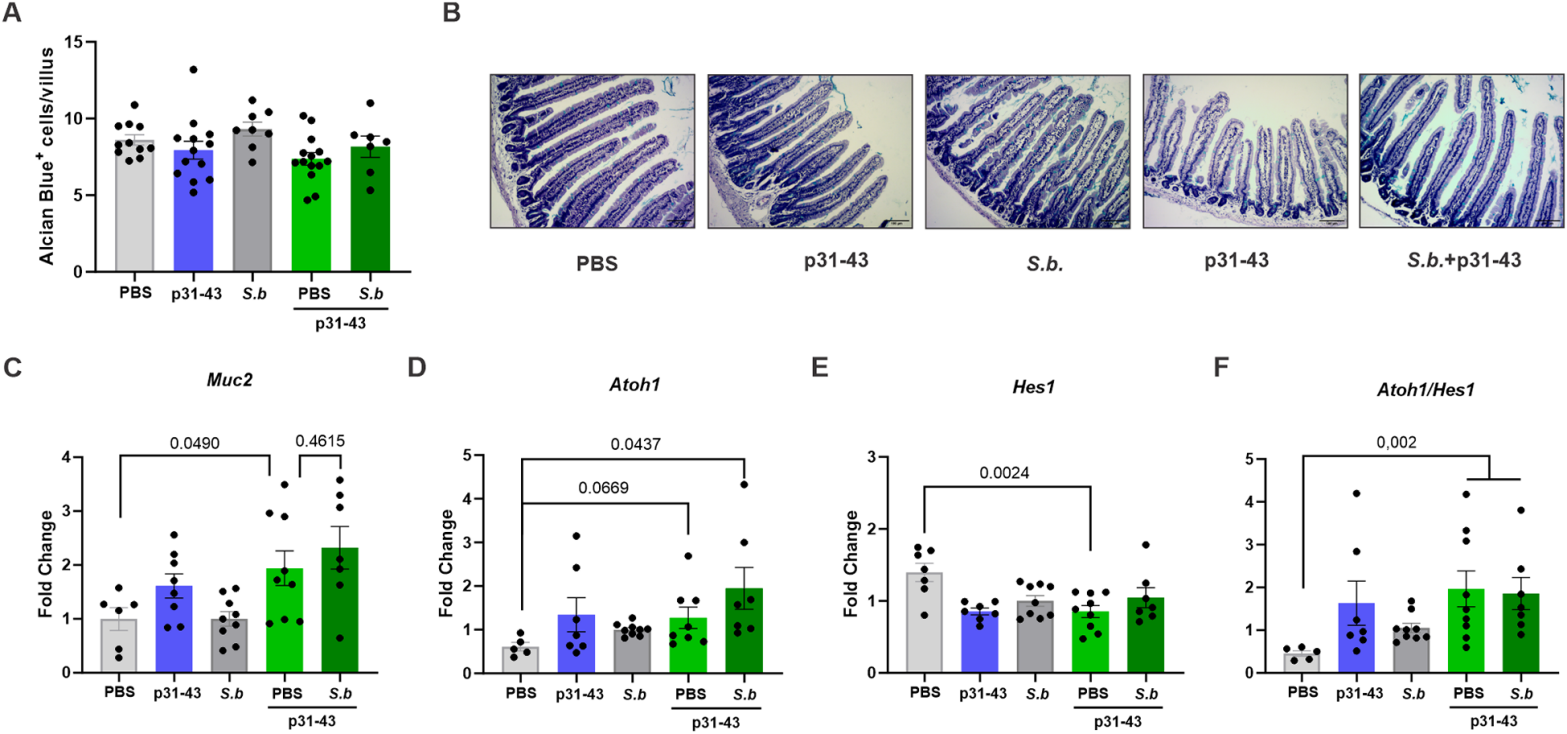
Histological and genetic analyses of goblet cells in the multiple-challenge p31–43 model. **(A)** The alcian-blue-positive cell count per villus. N = 7–14 mice. **(B)** representative microscopy images of small intestine alcian blue staining for goblet cell counting for each studied group. Real-time qPCR analysis of *Muc2* **(C)**, *Atoh1* **(D)** and *Hes1* **(E)** mRNA expression. **(F)** mRNA expression of *Atoh1/Hes1* ratio. All values of mRNA analysis were normalized to housekeeping mRNA expression (*Hprt*) and were expressed as a normalized fold increase relative to the *Sb*-treated group, using the 2^-△△Ct^ method. Data are from two independent experiments, N = 5–9 mice. The results are expressed as the mean ± SEM. The threshold for statistical significance was set to p < 0.05 in Student’s unpaired t-test.

Goblet cells have a key functional role in protection of the intestinal mucosa. To assess the epithelial response to a chronic inflammatory challenge, both the number and functional activity of Goblet cells were evaluated by counting alcian blue-stained cells in sections of the small intestine. There were no significant differences in goblet cell counts between the experimental groups (**Figure 6A**). However, *Muc2* gene expression was significantly upregulated in p31–43-treated mice, with a trend toward further increased expression in mice that also received *Sb* (**Figure 6C**).

Since the *Muc2* gene was found to be upregulated, we investigated the expression of two genes associated with the differentiation (*Atoh1*) and proliferation (*Hes1*) of secretory intestinal epithelial cells. RT-qPCR analysis of total mRNA from intestinal segments showed that the *Atoh1/Hes1* ratio was significantly higher in mice subjected to multiple p31–43 challenges, regardless of *Sb* administration (**Figure 6F**). These results indicate that repeated administration of p31–43 promotes the upregulation of *Muc2* expression and enhances the differentiation program of secretory epithelial cells, which indicate an epithelial response under chronic inflammatory conditions. In this setting, *Sb* administration slightly potentiated both *Muc2* expression and the associated secretory lineage differentiation pathway.

### Expression analysis of markers related to inflammation and tissue repair in the multiple p31–43 dose model

To evaluate the gene expression levels of inflammatory and tissue repair markers, total RNA extracted from small intestine samples collected 16 hours after the final p31–43 challenge was analyzed using RT-qPCR.

**Figure 7:**
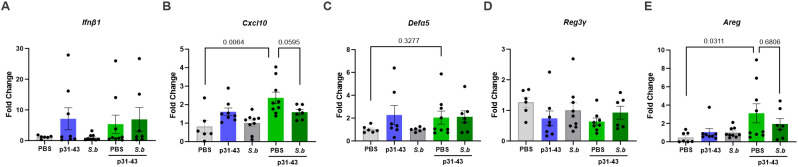
mRNA expression of genes associated with inflammatory and tissue-repair responses in the multiple-challenge p31–43 model. Real-time PCR analysis of **(A)** *Ifnβ1*, **(B)**. *Cxcl10*, **(C)**. *Defα5*, **(D)** *Reg3γ* and **(E)** *Areg* mRNA expression. All values were normalized to housekeeping mRNA expression (*Hprt*). All results were expressed as a normalized fold increase relative to the *Sb*-treated group, using 2^-△△Ct^ method. N = 6–9 mice. The results are expressed as the mean ± SEM. Results displayed in **(A, B, D)** were analyzed by Student´s unpaired t-test. Results displayed in **(C, E)** were analyzed by Mann– Whitney test. The threshold for statistical significance was set to p < 0.05 in Student’s unpaired t-test.

The mRNA expression level of *Ifnβ1* was elevated (albeit not significantly) in mice challenged with p31-43, with no changes observed after exposure to *Sb* alone (**Figure 7A**). As expected, repeated administration of p31–43 was associated with significant upregulation of *Cxcl10* gene expression. Notably, co-administration of *Sb* during the repeated p31–43 challenges resulted in the normalization of *Cxcl10* expression levels (p = 0.0595) (**Figure 7B**).

Analysis of the mRNA expression of the antimicrobial peptides *Def*α5 and *Reg3γ* did not reveal any significant difference in expression following multiple doses of p31-43 (irrespective of *Sb* coadministration) versus control groups (**Figures 7C, D**).

The mRNA expression levels of *claudin-2* and *claudin-4* did not differ significantly from one experimental group to another. The mRNA expression of *Areg* (a key gene in tissue repair) was significantly elevated in mice treated with p31–43 over a three-week period (**Figure 7E**). The group that received *Sb* concomitantly with repeated p31–43 challenges exhibited a slightly downregulation of *Areg* expression (**Figure 7E**).

Notably, *Sb* treatment effectively suppressed the upregulation of the pro-inflammatory chemokine *Cxcl10* driven by repeated p31-43–challenges.

#### *Saccharomyces boulardii* CNCM I-745 treatment prevented the increase in cell death and inflammasome activation triggered by multiple p31–43 challenges

**Figure 8:**
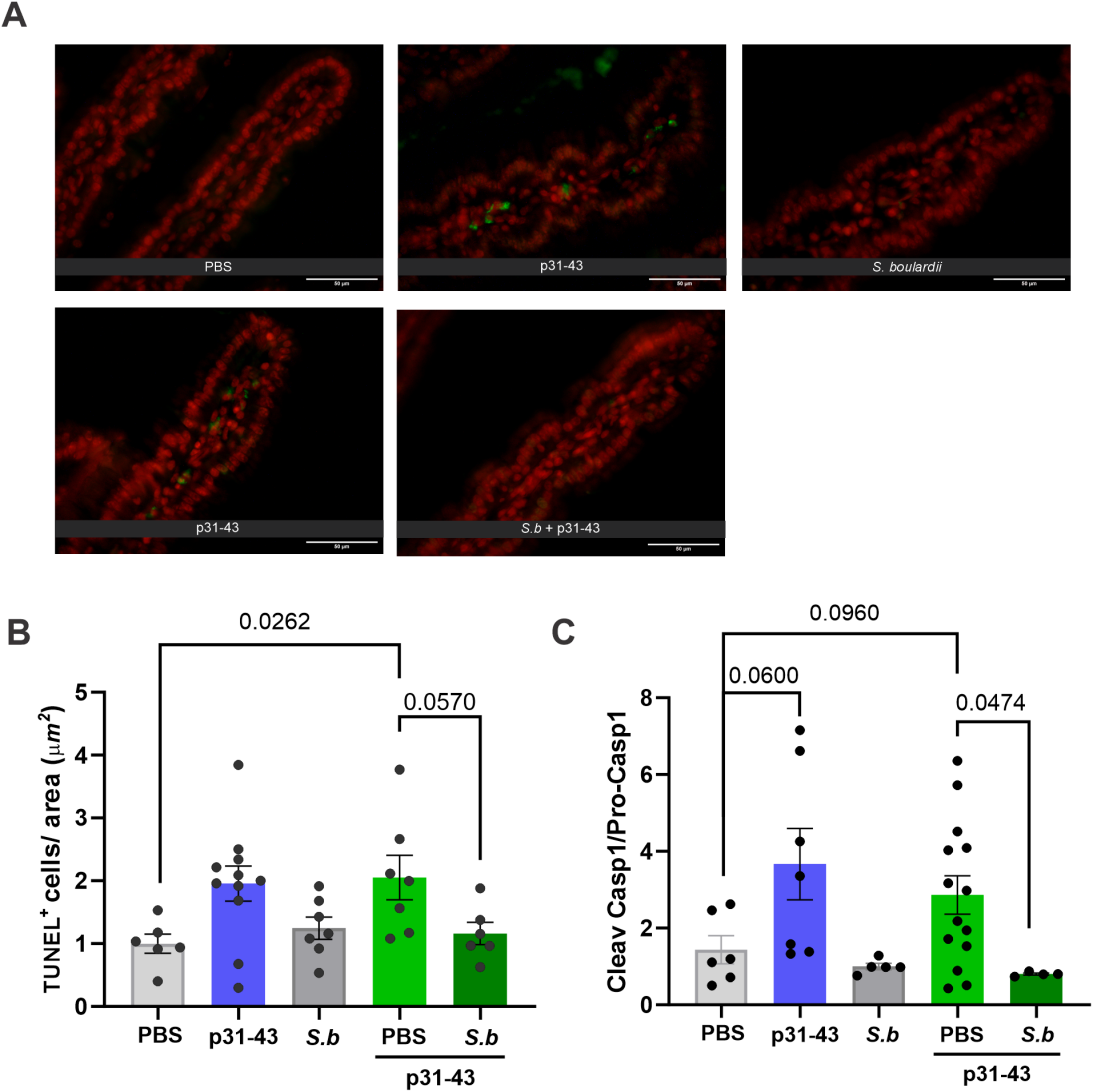
Analysis of caspase-1 activation and cell death in the proximal small intestine in the multiple-challenge p31–43 model. **(A)** Representative images of TUNEL staining of sections of proximal small intestine. Propidium iodide staining for the nuclei (red) of TUNEL-positive cells (green) **(B)** The TUNEL-positive cell counts per µm^2^ of *lamina propria* (normalized), expressed as the mean ± SEM. **(C)** Western blot analysis of cleaved caspase-1 and pro-caspase-1 quantification for protein extract of small intestinal fragments of individual mice. Data are from three independent experiments, N = 5–14 mice. Results are expressed as the mean ± SEM ratio between of cleaved caspase-1 and procaspase-1 band intensities. The threshold for statistical significance was set to p < 0.05 in Student’s unpaired t-test.

We next sought to determine whether *Sb* treatment could counteract the cell death in the small intestinal mucosa triggered by intragastric administration of p31-43. Treatment with p31–43 for three weeks induced a significant elevation in the number of TUNEL-positive cells. Remarkably, *Sb* treatment reversed this p31-43–induced increase in cell death (p = 0.057) (**Figure 8B**). The group treated with *Sb* alone did not differ significantly from the PBS control group.

To determine whether inflammasome activation occurs with multiple p31–43 challenges, caspase-1 activity was assessed by Western blot analysis of protein extracts from small intestine fragments. Inflammasome activation was indeed observed in mice challenged repeatedly with p31-43, whereas mice receiving *Sb* concomitantly exhibited a significantly lower level of caspase-1 activation (p = 0.0474) (**Figure 8C**). Mice receiving *Sb* alone also had a lower level of caspase-1 activation.

These striking findings suggest that *Sb* treatment inhibits cell death and inflammasome activation induced by repeated p31–43 challenges.

## Discussion

The results of the present study demonstrate that *Sb* protects against epithelial damage, inflammatory signaling, and regulated cell death in two complementary mouse models of sterile small intestinal enteropathy induced by the intragastric administration of the gliadin-derived peptide p31-43. Our laboratory’s mouse model of p31–43 challenge reproduces several histological features of celiac disease in the small intestine, i.e. a lower V/C ratio, a greater IEL count, and increased cell death and inflammasome activation (13, 15). Using both single-challenge and repeated-challenge p31–43 protocols, we show that *Sb* preserves intestinal architecture, normalizes pro-inflammatory chemokine expression, attenuates inflammasome activation, and limits epithelial cell death. Collectively, these findings identify *Sb* as a modulator of epithelial innate immune responses and mucosal homeostasis in the proximal small intestine.

Small intestinal inflammation compromises epithelial integrity and nutrient absorption, particularly when inflammation is persistent, and may promote systemic inflammatory consequences through increased gut permeability (17, 18). Although probiotics have been used to treat gastrointestinal inflammatory disorders, the corresponding mechanisms of action in the proximal small intestine have not been extensively characterized (1, 19). Most mechanistic studies have focused on colonic inflammation or infectious diarrhea, whereas sterile inflammation of the small intestine has received relatively little attention. The present study addressed this gap by evaluating *Sb* in a model driven by epithelial stress and innate immune activation, rather than microbial infection. In both the single-challenge and repeated-challenge protocols, *Sb* supplementation preserved the V/C ratio - a key indicator of epithelial integrity. Maintenance of epithelial architecture is essential for optimal absorptive and barrier functions (18). In the single-challenge model, *Sb* preserved crypt depth and so might have stabilized the proliferative compartment during the early phase of inflammatory stress. In the repeated-challenge model, *Sb* restored villus height; this is consistent with greater epithelial regeneration after sustained injury. These observations are in line with previous reports in which *Sb* promoted epithelial restitution and repair processes (7, 20).

The recruitment of inflammatory cells (and particularly IELs) into the epithelium is an early hallmark of small intestinal inflammation and is strongly associated with CXCL10 signaling (21). We observed that the administration of *Sb* normalized p31-43–induced *Cxcl10* upregulation and reduced IEL infiltration in both the single-challenge and repeated-challenge models. CXCL10 is induced downstream of IFN signaling and NF-κB activation (22). Notably, *Sb* has been shown to inhibit NF-κB activation in intestinal epithelial cells and immune cells (8, 23). Thus, the lower level of *Cxcl10* expression observed in our study probably reflects modulation of upstream innate signaling pathways in the epithelium. By limiting chemokine production, *Sb* might attenuate IEL recruitment and prevent the amplification of mucosal inflammation.

One of our study’s most significant findings was the attenuation of regulated cell death pathways by *Sb*. Previous research has demonstrated that p31–43 induces inflammasome activation and epithelial cell death in the small intestine (13, 15). Inflammasome activation results in caspase-1 cleavage and maturation of IL-1β and IL-18 (24, 25). Here, *Sb* markedly reduced caspase-1 activation in both single-challenge and repeated-challenge models, indicating suppression of inflammasome signaling. These findings are consistent with reports in which *S. boulardii* reduces NLRP3 inflammasome activation in experimental colitis models and in macrophage-based *in vitro* assays (26, 27).

In addition to inflammasome modulation, *Sb* reduced caspase-3 activation and the TUNEL-positive cell count; these results indicate that apoptosis was attenuated. The concurrent reduction in apoptosis suggests that *Sb* acts upstream of terminal cell death pathways - possibly by limiting epithelial stress signals and danger-associated molecular pattern release. Preservation of epithelial viability is critical for maintaining barrier integrity and preventing secondary inflammatory amplification (17).

Amphiregulin (encoded by *Areg* in the mouse) is an epidermal growth factor receptor ligand with an essential role in epithelial repair and tissue recovery (28). *Sb* enhanced the expression of *Areg* after a single p31–43 challenge, which suggests that repair mechanisms were activated. Repeated p31–43 challenges also induced *Areg* expression, and *Sb* did not enhance this response further; repair signaling might have reached the maximal level of activation. These findings are consistent with the hypothesis whereby probiotics not only suppress inflammatory pathways but also promote epithelial repair programs (7).

The mucus barrier is a critical component of the mucosal defense mechanism, and MUC2 is the predominant secreted mucin in the small intestine (29). Although the goblet cell count did not vary, repeated p31–43 exposure increased *Muc2* expression and shifted the *Atoh1/Hes1* ratio towards secretory lineage differentiation. ATOH1 is a master regulator of secretory cell differentiation, whereas HES1 promotes absorptive lineage commitment (30). The fact that *Sb* potentiated these responses to a moderate extent suggests that probiotic treatment supports epithelial adaptation under chronic inflammatory stress but does not induce pathological hyperplasia.

It is important to bear in mind that we studied a model of sterile inflammation. Consistently, we did not observe major changes in the mRNA expression of genes coding for antimicrobial peptides or tight junction components. In models of infection, *Sb* exerts its protective effects through toxin neutralization and pathogen binding (9–11). In contrast, the protective effects observed here probably reflect direct immunomodulatory and cytoprotective actions on epithelial signaling pathways.

Our results also indicate that *Sb*’s protective effects differed slightly in the single-challenge versus repeated-challenge p31–43 models (**Supplementary Table 1**). In the single-dose model, *Sb* acted primarily as a preventive modulator of early epithelial stress responses by preserving crypt architecture, limiting the rapid induction of inflammatory mediators, and preventing the initiation of cell death pathways. These observations suggest that *Sb* interferes with the earliest stages of epithelial sensing and innate immune activation triggered by p31-43. In contrast, *Sb* exerted a more pronounced effect on tissue remodeling and recovery during a three-week period of repeated p31–43 challenges; this was evidenced by greater villus height, normalization of IEL infiltration, and sustained control of inflammasome activation and epithelial cell death. Our findings indicate that *Sb* not only mitigates the onset of acute inflammatory damage but also supports long-term epithelial adaptation and recovery during chronic inflammation. The differences in *Sb*’s effects probably reflect the host’s transition from an early, transient, inflammatory response to a sustained state that requires the coordinated regulation of repair, differentiation, and immune homeostasis.

The present findings might be of relevance to the management of gluten-related disorders. The p31–43 peptide reproduces some of the features of the early, epithelial, innate stress response described in celiac disease, including inflammasome activation and epithelial damage (13, 14). Although the p31–43 challenge model does not recapitulate the adaptive, HLA-dependent immune component of celiac disease, it provides insight into early epithelial events triggered by gliadin peptides. The proinflammatory effects of p31–43 were also assessed *in vitro* in a Caco-2 cell model of the intestinal epithelium (31). Interestingly, the mTOR pathway activation induced by p31–43 challenge was blocked by incubating the Caco-2 cells with components released by the probiotic *Lactobacillus rhamnosus* GG. Hence, *Sb* and *Lactobacillus rhamnosus* might modulate p31-43’s pro-inflammatory effects through distinct mechanisms.

Our study of a murine model of intestinal inflammation had limitations. Although the model reflects innate-driven, sterile inflammation and mucosal damage in the small intestine, we did not analyze barrier permeability and the composition of the microbiota. Hence, data from functional assays (e.g. FITC-dextran assays of gut barrier permeability) are still needed. In order to better define the mechanisms underlying the *Sb*-mediated protection observed here, future studies should evaluate functional barrier integrity, immune cell phenotypes, and host–microbiota interactions.

In conclusion, *Saccharomyces boulardii* CNCM I-745 preserved intestinal architecture, suppressed pro-inflammatory chemokine expression, inhibited inflammasome activation, and reduced epithelial cell death in models of acute and chronic sterile inflammation of the small intestine. These findings identify *Sb* as a modulator of epithelial innate immunity and regulated cell death pathways in the proximal small intestine and indicate a potential role for this probiotic in protection against inflammation-induced enteropathy.

## Data Availability

The raw data supporting the conclusions of this article will be made available by the authors, without undue reservation.

## Supplementary material

Supplementary Table 1 Effects of *Saccharomyces boulardii* CNCM I-745 administration on small intestinal damage induced by single and repeated challenges with p31-43
