# Supplementary material for: Saccharomyces boulardii CNCM I-745 protects against small intestinal injury in a model of gliadin-peptide-induced sterile inflammatory enteropathy
Source: Front Immunol. 2026 Jul 6;17:1846961. doi: 10.3389/fimmu.2026.1846961 (PMC13381514; doi:10.3389/fimmu.2026.1846961)
Supplement: Supplementary Table 1 — Effects of Saccharomyces boulardii CNCM I-745 administration on small intestinal damage induced by single and repeated challenges with p31-43 [file Table1.docx]

**Supplementary Table 1. Effects of *Saccharomyces boulardii* CNCM I-745 administration on small intestinal damage induced by single and repeated challenges with p31-43**

| **Marker** | **The single-challenge model** | **The repeated-challenge model** |
| --- | --- | --- |
| V/C ratio | 🡹 (due to reduction in crypt depth) | 🡹 (due to an increase in villus height) |
| IELs count | 🡻 | 🡻 |
| Goblet cells count | *not tested* | no change |
| *Atoh1/Hes1* ratio | *not tested* | no change |
| TUNEL-positive cells | 🡻 | 🡻 |
| Cleaved caspase-3 | 🡻 | *not tested* |
| Inflammasome activation | 🡻 | 🡻 |
| *Cxcl10* expression | 🡻 | 🡻 |
| *Ifnb1* expression | 🡻 | no change |
| *Areg* expression | 🡹 | no change |
